# Heteromultivalent Ligand Display on Reversible Self-Assembled Monolayers (rSAMs): A Fluidic Platform for Tunable Influenza Virus Recognition

**DOI:** 10.1021/acsami.3c15699

**Published:** 2024-01-10

**Authors:** Yulia Sergeeva, Sing Yee Yeung, Börje Sellergren

**Affiliations:** Department of Biomedical Sciences and Biofilms-Research Center for Biointerfaces (BRCB), Faculty of Health and Society, Malmö University, 205 06 Malmö, Sweden

**Keywords:** virus recognition, H5N1, H7N9, IAV subtype selectivity, virus monitoring

## Abstract

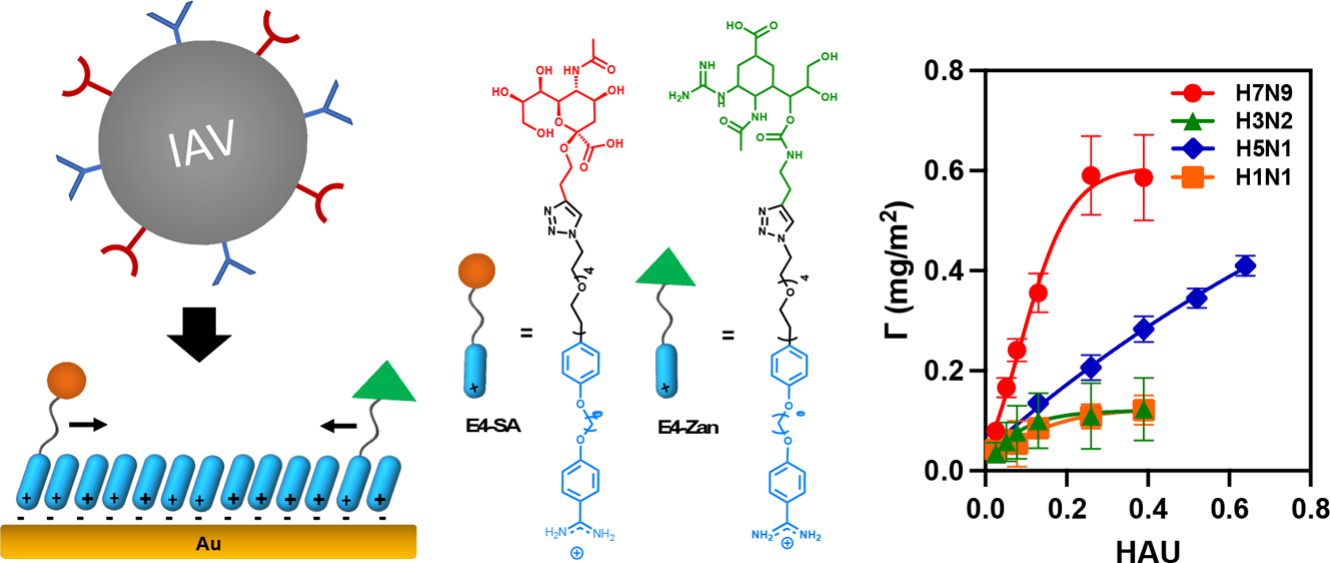

We report on the design of heteromultivalent influenza A virus (IAV) receptors based on reversible self-assembled monolayers (SAMs) featuring two distinct mobile ligands. The principal layer building blocks consist of α-(4-amidinophenoxy)alkanes decorated at the ω-position with sialic acid (SA) and the neuraminidase inhibitor Zanamivir (Zan), acting as two mobile ligands binding to the complementary receptors hemagglutinin (HA) and neuraminidase (NA) on the virus surface. From ternary amphiphile mixtures comprising these ligands, the amidines spontaneously self-assemble on top of carboxylic acid-terminated SAMs to form reversible mixed monolayers (rSAMs) that are easily tunable with respect to the ligand ratio. We show that this results in the ability to construct surfaces featuring a very strong affinity for the surface proteins and specific virus subtypes. Hence, an rSAM prepared from solutions containing 15% SA and 10% Zan showed an exceptionally high affinity and selectivity for the avian IAV H7N9 (*K*_d_ = 11 fM) that strongly exceeded the affinity for other subtypes (H3N2, H5N1, H1N1). Changing the SA/Zan ratio resulted in changes in the relative preference between the four tested subtypes, suggesting this to be a key parameter for rapid adjustments of both virus affinity and selectivity.

## Introduction

Influenza is a family of evolving viruses that yearly cause 1 billion cases of acute respiratory disease.^1,2^ Due to constant mutation, new subtypes of influenza virus strains emerge and can potentially cause a severe pandemic with high rates of mortality. In this context, the development of advanced sensors for the rapid diagnosis of the influenza virus is critical for disease control and the development of pandemic preparedness.^2^ The conventional diagnostic assays for influenza include polymerase chain reaction (PCR) tests that allow for the detection and subtyping of viruses with high sensitivity and specificity. However, these methods involve lengthy analytical procedures, specific equipment, and trained personnel.^3^ The second group of methods includes rapid diagnostic tests that can produce results within 15–20 min. However, these tests typically suffer from low sensitivity and are limited to circulating subtypes. In this context, biomimetic sensors employing glycans, particularly sialic acid (SA),^4^ as recognition elements are gaining attention.^5^ In these sensors, the detection relies on strong multivalent interactions between the immobilized sialic acid-terminated ligands and viral particles through interaction with the lectin hemagglutinin (HA), a trimeric surface protein that mediates viral binding to the target cells.^6^ As HAs from the human and animal subtypes of influenza A viruses (IAVs) bind to α2,6-linked and α2,3-linked SA on the cell surface, respectively, a careful design of the sialic acid ligands reflecting these structural features allowed discrimination between human and animal viruses.^7^ However, the rapid mutations in the HA binding domains may switch the receptor recognition preference,^8,9^ leading to a loss or reduction of the sensor specificity. Moreover, infectivity relies not only on the receptor binding protein HA but also on another IAV surface protein, the enzyme neuraminidase (NA).^10^ Ideally, sensors should therefore be sensitive to the presence of both surface proteins. NA, a 240 kDa tetrameric glycoprotein, catalyzes the cleavage of terminal sialic acids on the surface of the cells, helping an incoming virus to access the cell and a newly formed virus to escape it. The protein is the target of NA inhibitors (NAIs) that are currently used as emergency antiviral drugs.^11^ Due to the high mutation rate of IAVs, several NAI-resistant strains have emerged, some with enhanced infectivity. For this to occur, it has been found that the two surface proteins, HA and NA, need to act in concert. For instance, reduced NA activity following such mutations exerts selection pressure for strains with lower HA binding affinity or higher NA activity.^9^ The latter is promoted by the existence of a second SA binding site, juxtaposed to the enzyme active site.^12^ Strain-dependent differences in its affinity for SA therefore add to the variety of IAV binding properties to be elucidated by biomimetic sensor platforms.

The closest mimics of cellular membranes are lipid-based platforms such as supported lipid bilayers (SLBs), liposomes, lipid disks, or cubes.^13^ These exhibit mobile ligands that can diffuse laterally to optimize receptor binding and thereby promote strong multivalent interactions,^14^ often exceeding singular interactions by more than 3 orders of magnitude. Such systems are ideally suited for studying IAV host–cell receptor interactions and other heteromultivalent interactions, i.e., binding events involving multiple distinct ligands and/or receptors.^15,16^ Importantly, this allows us to identify weak affinity coreceptors that often escape detection in homogeneous assays. The development of more potent inhibitors also profits from such designs. Indeed, bioactive ligand-functionalized vesicles or membrane-covered nanoparticles hold promise as broad-spectrum biocompatible virus inhibitors.^17,18^

Recently, we reported on an air-stable and adaptable biosensing platform, reversible self-assembled monolayers (rSAMs), featuring strongly enhanced affinity and sensitivity toward deactivated IAVs and lectins.^19−21^ This sensing platform utilizes noncovalent amidinium-carboxylate ion pairs for building stable two-dimensional assemblies, akin to lipid bilayers but with a simple preparation process and fast on/off rates. Thus, benzamidine-terminated amphiphiles spontaneously assemble in a neutral or alkaline aqueous solution on alkanoic acid-functionalized thiol SAMs to form ordered monolayers with a tunable pH responsiveness.

We here demonstrate that these systems can be used as highly versatile abiotic platforms for sensing and studies based on heteromultivalent interactions. We show that mixed rSAMs based on three amphiphiles with head groups comprising SA and the NAI Zanamivir offer a tunable sensing platform to control IAV affinity and selectivity. Based on in situ ellipsometry (IES), infrared reflection adsorption spectroscopy (IRAS), and atomic force microscopy (AFM), we have investigated the structure and recognition properties of such rSAMs and demonstrated their use for discriminating between the IAV subtypes A(H5N1), A(H1N1), A(H3N2), and A(H7N9).

## Experimental Section

### Reagents

Sodium hydroxide (≥98% pure), HEPES (≥99.5% pure), and 4-mercaptobenzoic acid (MBA) were purchased from Sigma-Aldrich. Water used for the preparation of all solutions was purified with the PURELAB Chorus purification system (Veolia Water Technologies, Saint-Maurice. France). Recombinant N2 NA with Histidine Tag from Influenza A/canine/Illinois/11613/2015 (H3N2) (FR-1479), BPL-Inactivated Influenza A Virus, A/Anhui/01/2005(H5N1)-PR8-IBCDC-RG6 (FR-918), BPL-Inactivated Influenza A, A/Michigan/45/2015 (H1N1)pdm09 (FR-1506), BPL-Inactivated Influenza A Virus, A/Alaska/232/2015 (H3N2) (FR-1541), and BPL-Treated Influenza A, A/Anhui/1/2013 (H7N9) (FR-1283) were obtained from the International Reagent Resource, Influenza Division, WHO Collaborating Center for Surveillance, Epidemiology and Control of Influenza, Centers for Disease Control and Prevention, Atlanta, GA, USA. E2-OH, E4-SA, and E4-Zan were synthesized as described in our previous reports^19,21^ and in the Supporting Information (E4-Zan).

### Formation of Anchor Layers

The gold surfaces were prepared by electron beam (e-beam) evaporation of gold (2000 Å thickness) onto precleaned glass slides (76 mm × 26 mm × 1 mm) containing adhesive layers (25 Å) of titanium. Before thiol adsorption, the gold surfaces were treated with a plasma cleaner. The MBA SAMs were prepared by immersing the gold-covered substrate in 1 mM MBA in ethanol (99.5%) for 24 h followed by rinsing with a copious amount of ethanol and drying under a nitrogen stream.

### Ellipsometry Measurements

The amidine adsorption was monitored using a Rudolph thin-film ellipsometer (type 43603-200E, Rudolph Research, USA) operating at an angle of incidence of 68° and automated according to Cuypers et al.^22^ The light source was a xenon lamp, filtered to λ = 442.9 nm. The experiment was performed in HEPES buffer (10 mM, pH 8) at 25 °C and a constant stirring rate of 350 rpm. Before each measurement, the refractive index of the MBA gold substrate was determined by a 4-zone surface calibration in HEPES buffer (10 mM, pH 8).

### Formation of Amidine Layers

The mixture of amidines (2.5 mM, HEPES buffer) was added to the cuvette after achieving a stable baseline. The final total amidine concentration was 50 μM. Kinetics data was recorded until stabilization, and then the system was rinsed with HEPES buffer (10 mM; pH 8) for 300 s (11 mL/min). The thickness and the adsorbed mass were calculated using a three-layer substrate/film model using the refractive index for the liquid: 1.335. The effective complex refractive index for the rSAMs was assumed to be 1.45. A refractive index increment, d*n*/d*c*, of 0.22 mg/mL was used to determine the amount of rSAMs adsorbed.

### Interaction with N2NA and IAVs

After the formation of the amidine layer, the incremental amount of a solution of N2NA in HEPES buffer or inactivated IAVs in allantoic liquid was added to the cuvette in the concentration range 0.01 pM to 10 nM, and the changes in the thickness and the adsorbed mass were recorded for 2000 s for protein adsorption and 1200 s for virus adsorption.

### Infrared Reflection Absorption Spectroscopy

The measurements were carried out using a Nicolet 6400 spectrometer (Thermo Electron Corporation, USA) equipped with a liquid-nitrogen-cooled MCT-A detector operating at a resolution of 4 cm^–1^. Data were collected with a smart Saga accessory operating at an angle of incidence of 80°. The instrument was purged with compressed air before and during measurements. Each spectrum is the sum of 500 scans on the modified surfaces using an unreacted, cleaned gold substrate as a reference. Each spectrum was processed using OMNIC software and baselined corrected.

### AFM Measurements

The surfaces modified as described above were examined with a commercial atomic force microscope (MultiMode 8 SPM with a NanoScope V control unit, Bruker AXS) in air at room temperature in the PeakForce Tapping mode. Cantilevers SCOUT 70 RAI, NuNano with a nominal spring constant of 1.557 N m^–1^, and 63.38 kHz were employed. Analysis and processing of AFM images were performed using WSxN 5.0 Develop 8.2.5. Each substrate was scanned randomly at min 3 points.

## Results and Discussion

### System Design and Characterization

**Figure 1:**
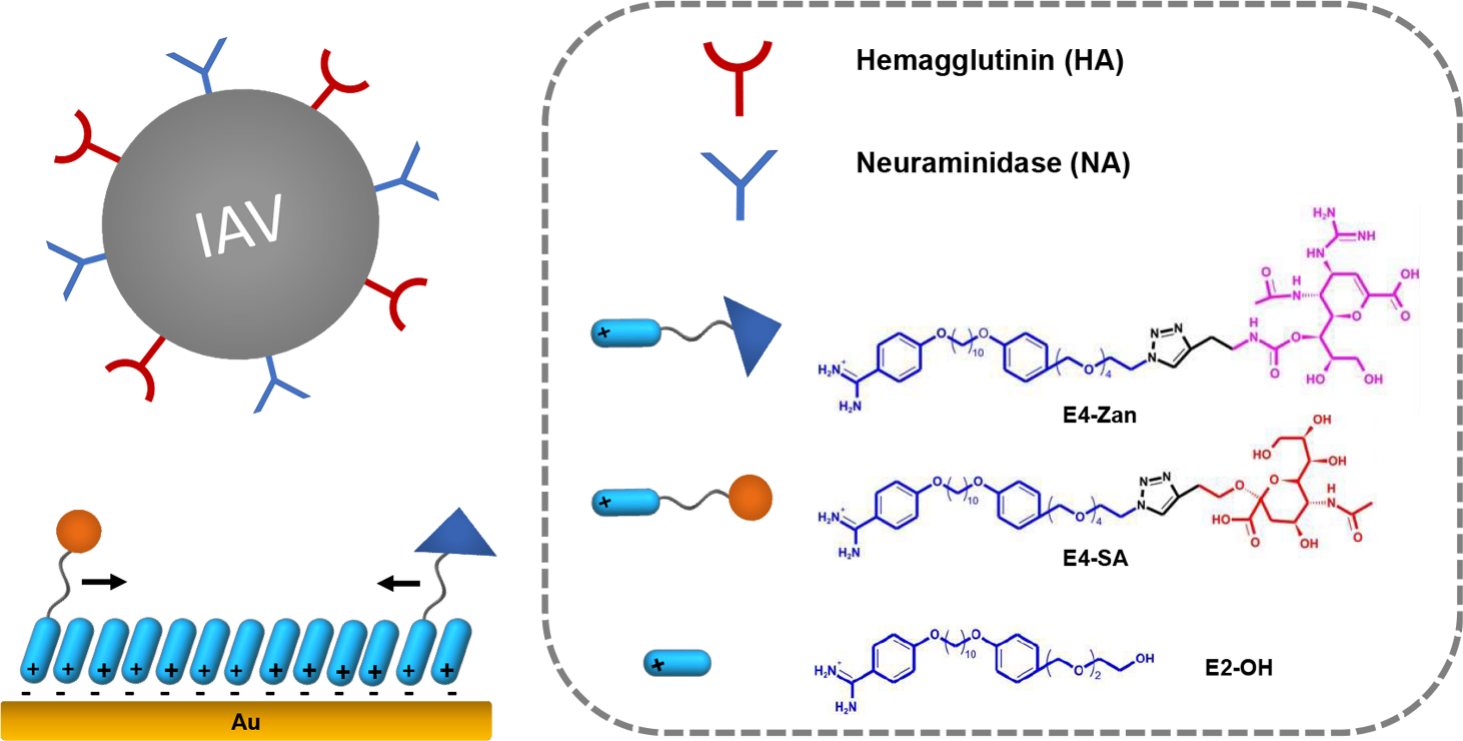
Schematic illustration of rSAM-based heteromultivalent receptors for IAV recognition.

Optimization of ligand-decorated SAMs demands attention to multiple factors governing the multivalent interactions with the receptor. Key parameters are the nature of the ligand, the length of the tether connecting the ligand headgroup and mesogen unit, and the surface density of ligand amphiphiles. Previously, we have demonstrated that optimal hemagglutinin binding to sialic acid rSAMs is achieved using a tether containing four ethylene glycol repeating units (E4-SA, Figure 1) and an SA ligand density of 15%.^21^

With this as a starting point, our aim was here to design a heteromultivalent receptor by introducing NAI Zanamivir (Zan) as a second ligand. Zan exhibits broad spectrum inhibition of NAs with IC_50_ values in the low or subnanomolar range.^10^ In order not to compromise the affinity of the ligand for the receptor, the mode of linking the ligand with the tether is crucial. The crystal structure of Zan bound to neuraminidase reveals the 7-hydroxy position to be solvent exposed and not involved in contacts with NA,^23^ a finding supported by the maintained antiviral activity of Zanamivir derivatized at this position.^24^ Exploiting this linkage mode E4-Zan was synthesized by copper-catalyzed cycloaddition between alkyne-modified Zan and the corresponding azide-terminated amidine^25^ following our previously reported protocols (see Supporting Information). Combined with E4-SA and E2-OH, the three amidines constitute the ternary amphiphile system used here to tune virus affinity and selectivity. This contrasts with a common design strategy to impart subtype specificity through the sialic acid-galactose linkage mode.^6^

### SA and Zan Amidines Form Mixed and Ordered rSAMs on Carboxylic Acid SAMs

**Figure 2:**
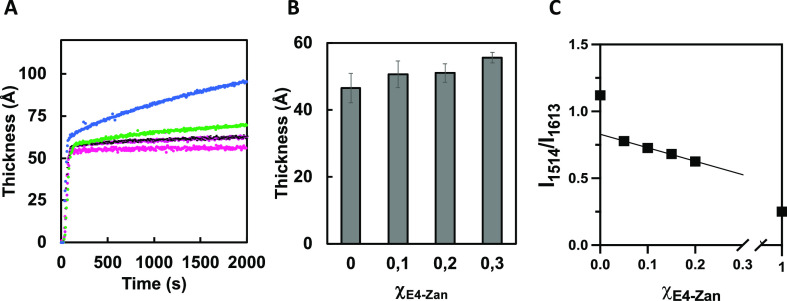
(A) Real-time change in film thickness upon immersion of MBA-modified gold substrates in ternary amphiphile mixtures in HEPES buffer (10 mM, pH 8). The molar ratio of E4-Zan and filler E2-OH was varied while keeping the E4-SA mole fraction fixed at χ_E4-SA_ = 0.15 (χ_E4-Zan_ = 0: pink trace; χ_E4-Zan_ = 0.1; 0.2: black/red trace; χ_E4-Zan_ = 0.3: green trace) with exception for χ_E4-SA_ = 1.00 (light blue trace). (B) Composition and thickness of the mixed rSAMs. Values are expressed as means ± SD, *n* = 3. (C) Ratio of the signal intensities of the E4-Zan characteristic C=C stretch signal at 1514 cm^–1^ to the benzamidine signal at 1613 cm^–1^ versus χ_E4-Zan_.

**Figure 3:**
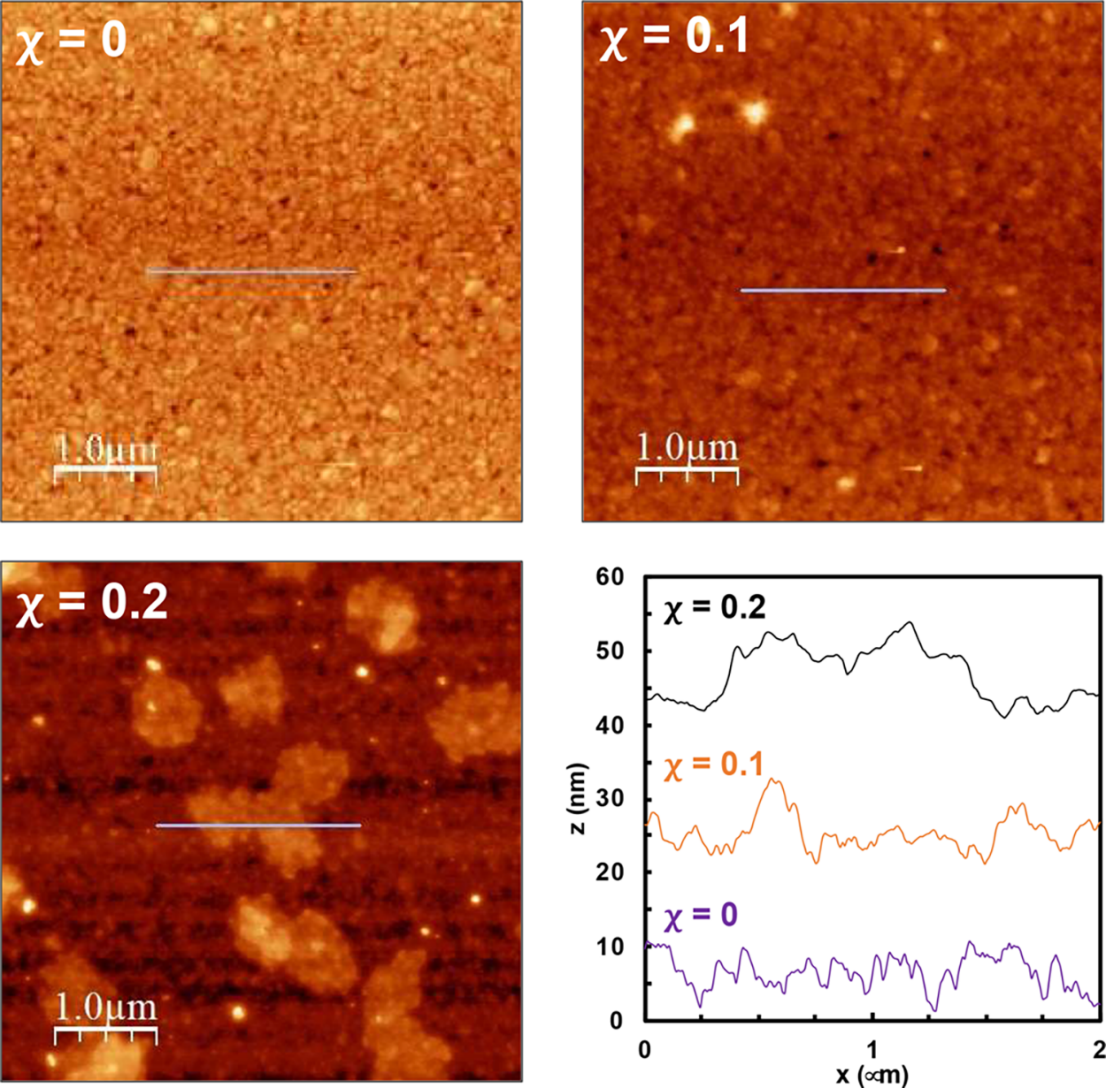
AFM topography and height profile of layers formed from E2-OH, E4-SA, and E4-Zan with χ_E4-SA_ = 0.15 and χ_E4-Zan_ = 0, 0.1, and 0.2 in the adsorption solution. The corresponding roughness values were *R*_rms_ = 2.5 ± 0.2, 2.6 ± 0.4, and 4.5 ± 0.6 nm.

In view of the diverse affinities for Zan and SA among different IAV strains, our plan was to use the Zan/SA ligand ratio as a parameter for the design of IAV strain selective surfaces. Based on our previous report demonstrating optimal sialic acid coverages with respect to hemagglutinin binding of 15%,^21^ a series of mixed rSAMs were prepared by immersing MBA-modified gold surfaces in pH 8 HEPES buffer containing different mole fractions of E4-Zan and filler E2-OH while keeping the E4-SA mole fraction fixed at χ_E4-SA_ = 0.15. Layer composition and molecular order and orientation of the rSAM amphiphiles were first evaluated by ISE, IRAS, and AFM (Figures 2, 3, S1, and S2).

ISE measures refractive index- and film thickness-sensitive changes in the polarization when light is reflected from a surface. The ellipsometric angles Δ and Ψ correlate with the phase shift and amplitude ratio of the s- and p-components of the reflected light and are used to estimate the film thickness and mass in real time. Figures 2A and S2 show the average film thicknesses during the adsorption of binary (Figure S2) or ternary (Figure 2A) amphiphile mixtures in pH 8 HEPES buffer (total concentration = 50 μM). Immediately after injection, steep thickness increases were observed that leveled off at ca. 55 Å within 1 min, in good agreement with our previous report.^21^ In the presence of the Zan-amphiphile, this was followed by a slower adsorption phase presumably due to weak adsorption of a second layer.^26^ This is particularly pronounced for the single-component monolayer of E4-Zan where the thickness leveled off at values exceeding 100 Å. Given the close agreement with the geometrical end-to-end distance of two stretched out E4-Zan molecules, we infer this to the formation of two layers of Zan with an upright orientation of the layer amphiphiles. Rinsing appeared to remove most of the second loose layer, as indicated by the thickness after rinsing results in Figure 2B. The increase in film thickness after rinsing upon increased E4-Zan moreover supports a successful incorporation of this amphiphile in the rSAM.

IRAS was then used to further confirm the nature of the mixed rSAMs. The spectra of the rSAMs (Figure S1) were compared with respect to features, informative of amphiphile stoichiometry as well as order and orientation of the molecules. Figure S1 reveals the significant peaks of the anchor SAM (MBA) and the three-component rSAMs. The former was distinguished by the C=C stretch band at 1585 cm^–1^ and the COO^–^ stretch at 1405 cm^–1^, whereas the latter comprised the C–H stretch bands of the alkyl chains at 2929, 2848–2863 cm^–1^, the sharp and intense aromatic C=C stretch signals of the bolaamphiphiles at 1613, 1514, 1497, and 1472 cm^–1^, the C–O–C stretch bands at 1243–1255 cm^–1^, the aliphatic ether band at 1188 cm^–1^, and the weak C–H out-of-plane bending signal at 841 cm^–1^. The intense C=C stretch bands having transition dipole vectors oriented along the 1,4-axis of the benzene rings relative to the bands representing perpendicular transitions at 841 cm^–1^ are in line with our previous reports and indicate a near upright position of the layer amphiphiles.^26^

We then searched for signals confirming the presence of E4-SA and E4-Zan. In agreement with our previous report, the presence of E4-SA could be confirmed in the binary component rSAMs (χ_E4-SA_ = 0.15; χ_E4-Zan_ = 0) by increased intensities of the broad band at 3350 cm^–1^ (H-bonded OH and monosubstituted amide) and the aliphatic ether band at 1188 cm^–1^ (C–O–C) compared to the spectrum of the filler alone (χ_E2-OH_ = 1). Increasing the E4-Zan component revealed more subtle changes in the spectra. This included a slight but notable increase in the intensity of the 3350 cm^–1^ band accompanied by overlapped bands assigned to the guanidine group at 3130 cm^–1^. Furthermore, increased intensities of signals corresponding to Zan at 1715 cm^–1^ and a decreased intensity of the C=C stretch signal at 1514 cm^–1^ versus the 1613 cm^–1^ signal (Figure 2C) confirmed an increased incorporation of E4-Zan with an increasing solution mole fraction.

AFM images of the rSAMs performed in the peak force tapping mode are seen in Figure 3. The image of the rSAM prepared in the absence of E4-Zan (χ_E4-Zan_ = 0) was relatively featureless with a roughness (*R*_rms_ = 2.5 nm). rSAMs formed in the presence of E4-Zan featured higher roughness values with *R*_rms_ = 4.5 nm for the rSAM with χ_E4-Zan_ = 0.20. These rSAMs featured distinct nanosized domains that we previously attributed to the clusters of the ligand-terminated amidines with the shorter domains primarily populated by the filler amidine E2-OH. The results suggests that E4-Zan has particularly strong tendency to form clusters, presumably due to the zwitterionic nature of the headgroup.

### NA and HA Bind with High Affinity to Mixed rSAMs

**Figure 4:**
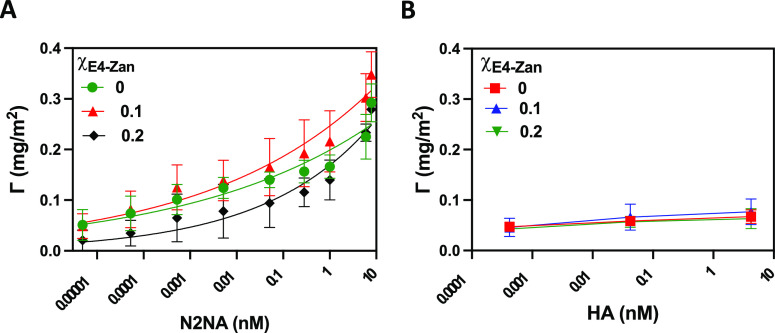
NA (A) and HA (B) binding isotherms on rSAMs formed at constant mole factions of E4-SA, χ_E4-SA_ = 0.15 and various mole fractions of E4-Zan, χ_E4-Zan_ = 0–0.2. Values are expressed as means ± SD, *n* = 3.

Having the ternary mixed rSAM at hand, the next goal was to verify that both ligands were accessible for binding to the IAV surface proteins. We therefore exposed the different mixed rSAMs to HA or N2NA in separate experiments and monitored the thickness and adsorbed mass by ISE (Figures S3–S5). Figure 4 shows the adsorbed mass after 2000 s of adsorption time versus the concentrations of N2NA (Figure 4A) and HA (Figure 4B) on rSAMs prepared using different mixing ratios. First, we note that significant protein binding could be observed at subpicomolar concentrations reflecting the overall high affinity of the mixed rSAMs for the two proteins. Moreover, none of the proteins required Zan for binding to occur, a nonsurprising finding given that SA is the natural ligand for HA and the documented presence of a second sialic acid binding site in N2NA.^12^ Nevertheless, a contrasting behavior of the two proteins was observed in response to the addition of E4-Zan. Whereas HA showed only a minor dependence on the E4-Zan ratio, the adsorption of N2NA responded distinctly to this parameter.

The rSAM prepared from solutions with χ_E4-SA_ = 0.15 and χ_E4-Zan_ = 0.10 showed slightly higher amounts adsorbed among the rSAMs, and this composition was therefore selected for further studies of virus recognition. Fitting the corresponding curve to a cooperative Hill equation resulted in a dissociation constant of *K*_d_ = 62 ± 9 pM, a value in the same range as the lectin affinity we reported previously.^21^ Furthermore, the rSAMs prepared from the highest molar ratio of E4-Zan (χ_E4-Zan_ = 0.20) showed the lowest NA affinity. As in our previous study on E4-SA rSAMs, this was accompanied by the formation of >100 nm sized clusters (Figure 3), possibly composed of less mobile amphiphiles.

### rSAMs Exhibit Tunable and Subtype-Specific Affinity for IAVs

**Figure 5:**
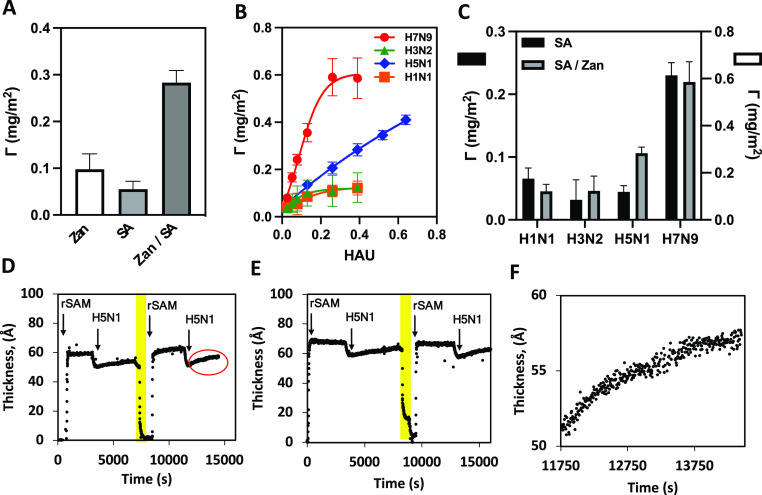
(A, C) Adsorbed amount, Γ, of A(H5N1) (A) or IAV subtypes, as indicated (C) at 0.4 HAU virus titer for rSAMs made of E2/E4-SA/E4-Zan with the following nominal compositions: Zan: χ = 0.75/0/0.25; SA: χ = 0.75/0.25/0; Zan/SA: χ = 0.75/0.15/0.10. (B) Adsorbed amount, Γ, on rSAMs made of E2/E4-SA/E4-Zan (χ = 0.75/0.15/0.10) upon addition of incremental amounts of inactivated H5N1 (blue), H7N9 (red), H1N1 (orange), and H3N2 (green). Values are expressed as means ± SD, *n* = 3. (D–F) Film thickness versus time measured by ISE for repeated adsorption of H5N1 (0.4 HAU) on the SA (D, F) and Zan (E) rSAMs in parts A–C. The rinsing step with pH 3 buffer is highlighted in yellow.

To assess whether rSAMs display type-specific IAV affinity, we evaluated the binding of four inactivated IAV strains including A(H5N1), A(H3N2), A(H1N1)pdm09, and A(H7N9) in allantoic liquid provided by the WHO. Focusing on the bird flu variant A(H5N1), our first goal was to assess whether the two ligands acted in concert to enhance the binding affinity and specificity. rSAMs based on single ligands (SA or Zan) or both ligands (Zan/SA) were therefore compared, keeping the ligand density (χ = 0.25) and virus titer (0.4 HAUs) fixed. Figure 5A convincingly demonstrates the cooperative effect of combining both ligands in the rSAM. Whereas the single ligand rSAMs SA and Zan showed binding uptakes of less than 0.1 mg/m^2^, the ternary rSAM Zan/SA exhibited a nearly 3-fold higher uptake. This suggests the simultaneous involvement of both HA and N2NA in the adhesion on these surfaces.

Titration experiments were then carried out on Zan/SA rSAMs under conditions identical to those used when testing surface protein binding (Figure 4) followed by plotting the limiting film thickness versus virus concentration (Figure 5B). The resulting binding curves for the different IAV strains revealed widely different affinities and uptakes. A(H1N1) is the strain binding with the weakest affinity to this rSAM, a result in agreement with literature reports showing a reduced sensitivity of A(H1N1)pdm09 to Zan.^27^ This is in strong contrast with the high response observed for A(H7N9). Fitting this curve to a cooperative Hill equation resulted in an exceptionally high affinity with a *K*_d_ = 11 ± 1 fM. A(H5N1) was the second strain responding distinctly to increasing concentrations of E4-Zan. This binding curve lacked curvature, precluding assessment of the binding affinity. Collectively, the binding of A(H5N1) and A(H7N9) is in agreement with the susceptibility of these strains to inhibition by Zanamivir.^28^ In view of the striking IAV subtype discrimination shown by the ternary rSAM, we decided to finally compare the IAV adsorption on this surface with the one lacking E4-Zan at a virus titer of 0.4 HAU (Figure 5C). Among the three subtypes tested, A(H5N1) was the subtype showing the strongest adsorption on the rSAM featuring E4-Zan, whereas the rSAM lacking E4-Zan showed an apparent preference for A(H1N1). This highlights the possibility of engineering subtype selectivity by fine-tuning the rSAM ligand composition.

Relying exclusively on monosaccharide ligands, the approach is distinctly different from the common exploitation of di- or trisaccharide ligands and SA-Gal linkage modes to achieve binding selectivity. Moreover, the pH-switchable surface modification allows repeated use of one single sensor surface for multiple consecutive measurements (Figure 5D,E).

## Conclusions

Lipid assemblies (e.g., SLBs, liposomes, lipid disks, etc.) featuring mobile ligands have so far been extensively studied for promoting heteromultivalent interactions, i.e., referring to binding events involving multiple distinct ligand–receptor pairs.^29^ As exemplified by plasma membrane-cloaked nanoparticles, such systems have demonstrated impressive potency as broad spectrum IAV inhibitors^18^ and for the elucidation of the role of weak affinity coreceptors^16^ that often escape detection in one-component assays. Despite this impressive performance, lipid assemblies are fraught with often lengthy and complex preparation protocols, lack of surface restorability, and limited stability. A versatile abiotic alternative to SLBs is offered by bola amphiphile-based rSAMs with the ability to spontaneously form densely packed monolayers on charged substrates. As demonstrated in this report, rSAMs are also an excellent platform for rapid tuning of heteromultivalent interactions.

Simply changing the ligand composition and ratio is a simple approach to influence the binding affinity as well as the selectivity for IAV subtypes. We believe the latter effect can be ascribed to a combination of different SA affinities, activities, and organization of the two surface proteins as well to differences in virus shapes and softness.^30^ These features are likely to require a dynamic sensor platform for being measurable. With IAV affinities in the femtomolar range and, a first of its kind, ligand ratio-dependent subtype preference, this study presents a practical way to engineer virus receptors for both analytical and medical uses. Analytical use can be envisaged in the form of readily tunable and restorable optical or electrochemical sensors for infection control or real-time environmental monitoring. Beyond virus recognition, the rSAM model may aid the deciphering of multivalent interactions in general and provide potent multivalent receptors and inhibitors^31^ for both molecular and cellular^32^ targets.

## Supporting Information Available

Reagents, apparatus and methods, synthesis of E4-Zan, and supporting characterization data (PDF)
