# Supplementary material for: Heteromultivalent Ligand Display on Reversible Self-Assembled Monolayers (rSAMs): A Fluidic Platform for Tunable Influenza Virus Recognition
Source: ACS Appl Mater Interfaces. 2024 Jan 10;16(3):3139–46. doi: 10.1021/acsami.3c15699 (PMC10811624; doi:10.1021/acsami.3c15699)
Supplement: Supplementary file 1 — am3c15699_si_001.pdf [file am3c15699_si_001.pdf]

Supporting Information

Hetero-multivalent ligand display on reversible self-assembled monolayers (rSAMs):

A fluidic platform for tunable influenza virus recognition

Yulia Sergeeva[1*], Sing Yee Yeung[1,2], Börje Sellergren[1*]

1) Department of Biomedical Sciences and Biofilms-Research Center for Biointerfaces (BRCB), Faculty of Health and Society, Malmö University, 205 06 Malmö, Sweden.

2) Present address: PYC Therapeutics, 6 Verdun Street, Nedlands, WA 6009, Australia

* Email:yulia.sergeeva@mau.se; borje.sellergren@mau.se

Table of contents

1 Experimental section 2

1.1 Reagents 2

1.2 Apparatus and methods 2

1.3 Procedures 3

2 References 4

3 Supporting figures 5

1 Experimental section

1.1 Reagents

All solvents were purchased from Acros Organics (Geel, Belgium) unless otherwise stated. Acetonitrile (ACN) was obtained from Merck (Darmstadt, Germany). Ethanol (99.5%) was obtained from CCS Health Care (Borlänge, Sweden). (4-(2-hydroxyethyl)-1-piperazineethanesulfonic acid (HEPES) and NaCl were obtained from VWR Chemicals (Leuven, Belgium). $MgSO_4$, anhydrous was purchased from JT Baker (Japan). Deionized water was used for chemical reactions. All other reagents were purchased from Sigma Aldrich (Sweden) or Merck (Sweden) and used as supplied unless otherwise stated. E2-OH, E4-SA and amidine azide 1 (Scheme 1) were synthesised as described in our previous reports.[1],[2] Zan-alkyne 2 (Scheme 1) was synthesised as previously reported by Fraser et al. The protected Zan precursor was obtained as a generous gift from the Fraser lab.

1.2 Apparatus and methods

Thin layer chromatography (TLC) was carried out using Merck aluminium backed sheets coated with 60F254 silica gel. Visualization of the silica plates was achieved using a UV lamp (max = 254 nm), and/or 5% ethanolic H_2SO_4.

HPLC analysis was carried out on a Waters 2695 Alliance HPLC system equipped with autosampler, inline degasser, Waters 2996 PDA detector and MassLynx 4.0 software, using a Phenomenex Luna C18(2) column (4.6 mm (i.d.) x 150 mm, 5 µm, 110 Å) and a guard column (4.6 x 20 mm) at ambient temperature. The mobile phase, as indicated in the procedure (vide infra), was pumped at a flow rate of 1.0 mL min^{-1}.

Flash column chromatography was carried out using Sigma Aldrich silica gel (Merck grade 9385, 60 Å). Reversed phase column chromatography was performed using an Agilent Bond Elute C18 column. The mobile phase used is as specified in the procedure (vide infra).

Proton and carbon nuclear magnetic resonance spectra were recorded using an Agilent (Varian) Mercury 400 MHz instrument operating at 400 or 101 MHz and evaluated using Mestre Nova software. Chemical shifts (δ) are reported in parts per million (ppm) with respect to tetramethylsilane (TMS) using the manufacturers indirect referencing method. All chemical shifts are quoted on the δ scale in ppm using residual solvent as the internal standard. (^1H NMR: CD_3OD = 4.87; and ^{13}C NMR: CD_3OD = 49.0). Coupling constants (J) are reported in Hz with the following splitting abbreviations: s = singlet, d = doublet, t = triplet, q = quartet, quin = quintet, and m = mutiplet.

Low resolution mass spectra (LRMS) were conducted using a Waters ZQ2000 MS system with 2795 LC and 2996 PDA. High resolution mass spectra (HRMS) were recorded by MALDI-MS analysis performed on a hybrid MALDI LTQ Orbitrap XL (Thermo Fisher Scientific, Germany) instrument. Nominal and exact m/z values are reported in Daltons.

1.3 Procedures

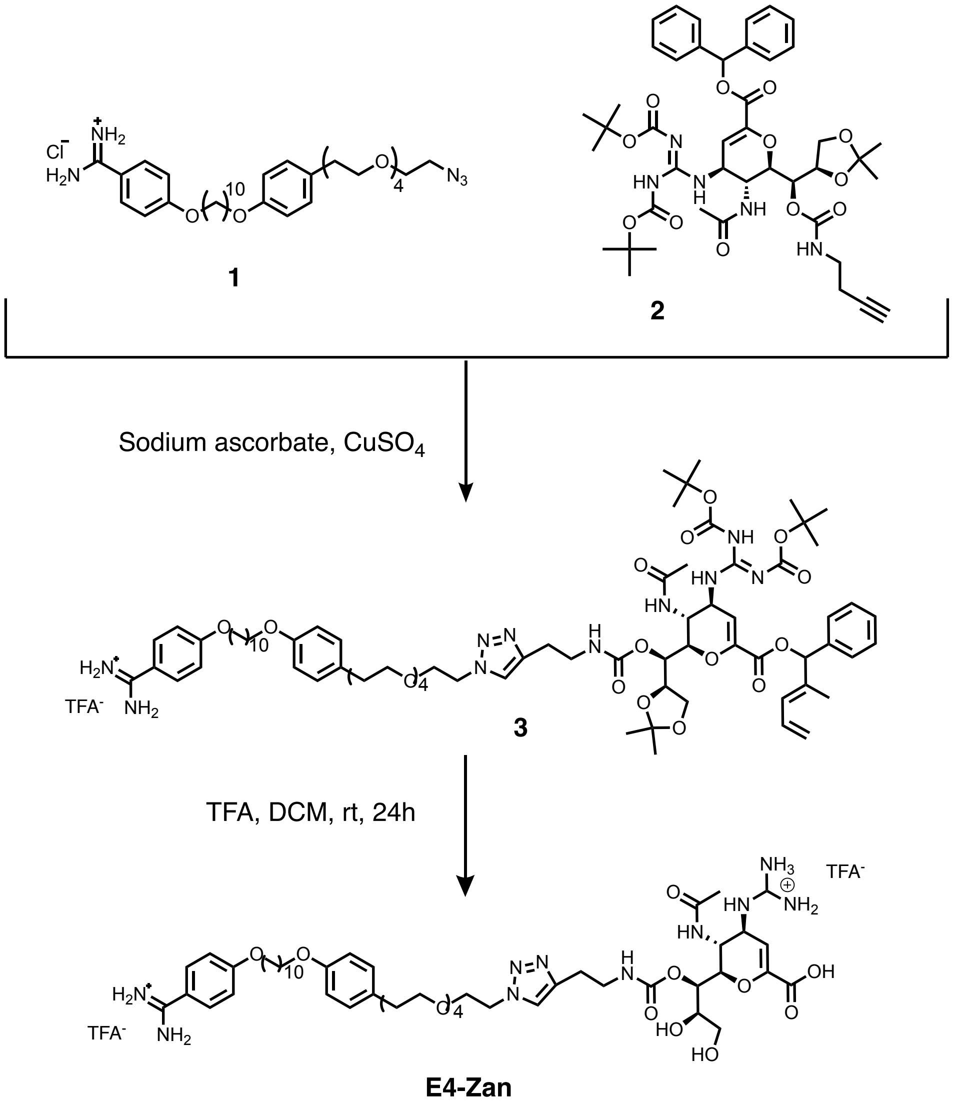

Scheme 1. Reaction steps in the synthesis of E4-Zan

E4-Zan from coupling of 1 and 2 and deprotection of 3

Amidine azide precursor 1 (37 mg, 0.06 mmol, 1 eq), Zan-alkyne 2 (50 mg, 0.06 mmol, 1 eq), sodium ascorbate (21 mg, 0.11 mmol, 1.8 eq) and copper (II) sulphate (15 mg, 0.06 mmol, 1 eq) in water/2-butanol (1:4, 10 mL) was sonicated and stirred at 40°C for 4 hrs. The reaction mixture was concentrated *in vacuo* and purified using C18 flash chromatography (10-100% MeCN, 0.1% TFA in H_2O). The purified fractions were then concentrated *in vacuo* at 40°C and the residual water was lyophilized to give the TFA salt of protected Zan terminated amphiphile 3 as an amorphous white powder (43 mg, 46 %). This was directly deprotected by treatment with TFA as follows.

To a flask containing the appropriate protected zanamivir dimer (43 mg) was added DCM (1 mL) and dry TFA (1 mL) and left overnight under stirring. The DCM and TFA were thereafter removed by distillation under reduced pressure. The residual was dissolved in 1:1 DMSO/water (2 mL) and purified by reverse phase chromatography using a gradient elution of 30% MeCN/water to 100% MeCN over 15 column volumes. E4-Zan was then obtained as a white solid after freeze drying (15.3 mg, 40%).

^{1}H-NMR (400 MHz, CD_3OD after D2O exchange) δ 9.06 (s, 1H), 8.63 (s, 1H), 7.93 (d, 2H), 7.76 (s, 1H), 7.71 (d, 2H), 7.52 (d, 1H), 7.07 (d, 2H)), 7.03 (d, 2H), 6.73 (d, 2H), 5.64 (d, 1H), 4.78 (d, 1H), 4.38 (t, 2H), 4.30 (d, 1H), 4.22 (s, 1H), 4.00 (t, 2H), 3.96-3.87 (m, 2H), 3.82 (t, 2H), 3.71 (t, 3H), 3.49-3.22 (m, 18H), 3.16 (m, 1H), 3.08 (t, 2H), 2.60 (p, 2H), 2.48 (p, 2H), 2.38 (p, 2H), 2.25 (p, 2H), 1.72 (s, 3H), 1.54-1.68 (m, 5H), 1.30-1.18 (m, 12H)

HRMS (*m*/*z*): $[M]^{2+}$ calcd for $C_{50}H_{77}N_{10}O_{14}^{2+}$, 1042.56, found 1042.02

2 References

(1) Yeung, S. Y.; Mucha, A.; Deshmukh, R.; Boutrus, M.; Arnebrant, T.; Sellergren, B. Reversible Self-Assembled Monolayers (rSAMs): Adaptable Surfaces for Enhanced Multivalent Interactions and Ultrasensitive Virus Detection. *ACS Cent Sci* **2017**, *3* (11), 1198-1207. DOI: 10.1021/acscentsci.7b00412.

(2) Yeung, S. Y.; Sergeeva, Y.; Dam, T.; Jonsson, P.; Pan, G.; Chaturvedi, V.; Sellergren, B. Lipid Bilayer-like Mixed Self-Assembled Monolayers with Strong Mobility and Clustering-Dependent Lectin Affinity. *Langmuir* **2019**, *35* (24), 8174-8181. DOI: 10.1021/acs.langmuir.9b01452.

3 Supporting figures

A

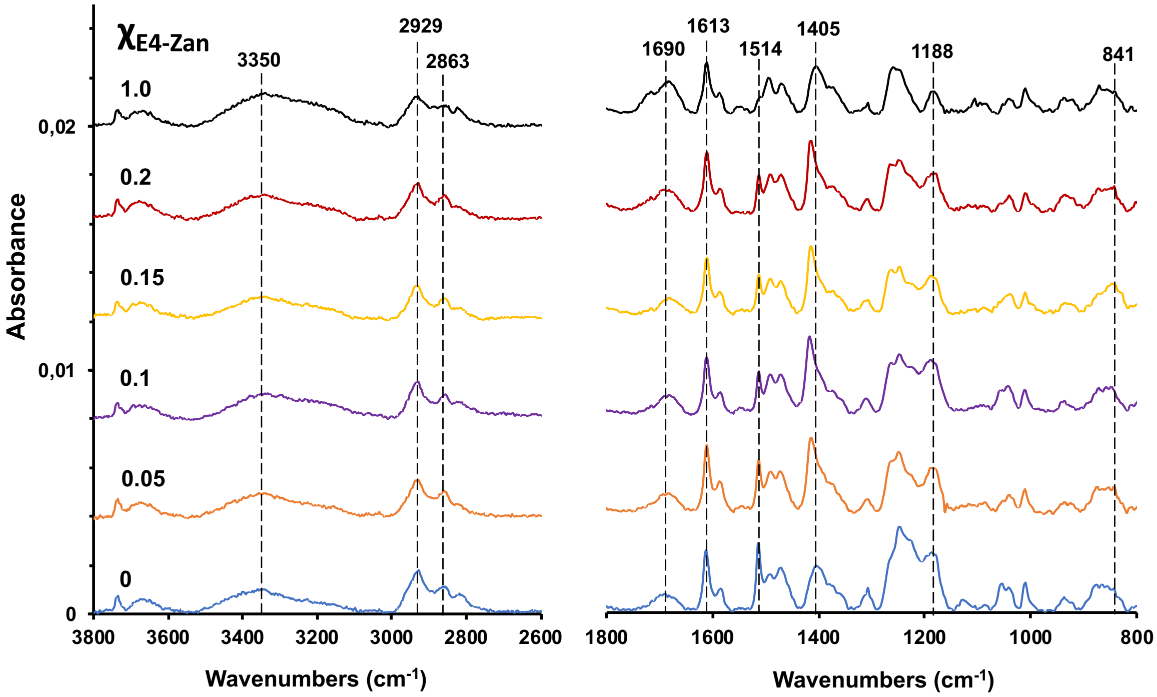

B

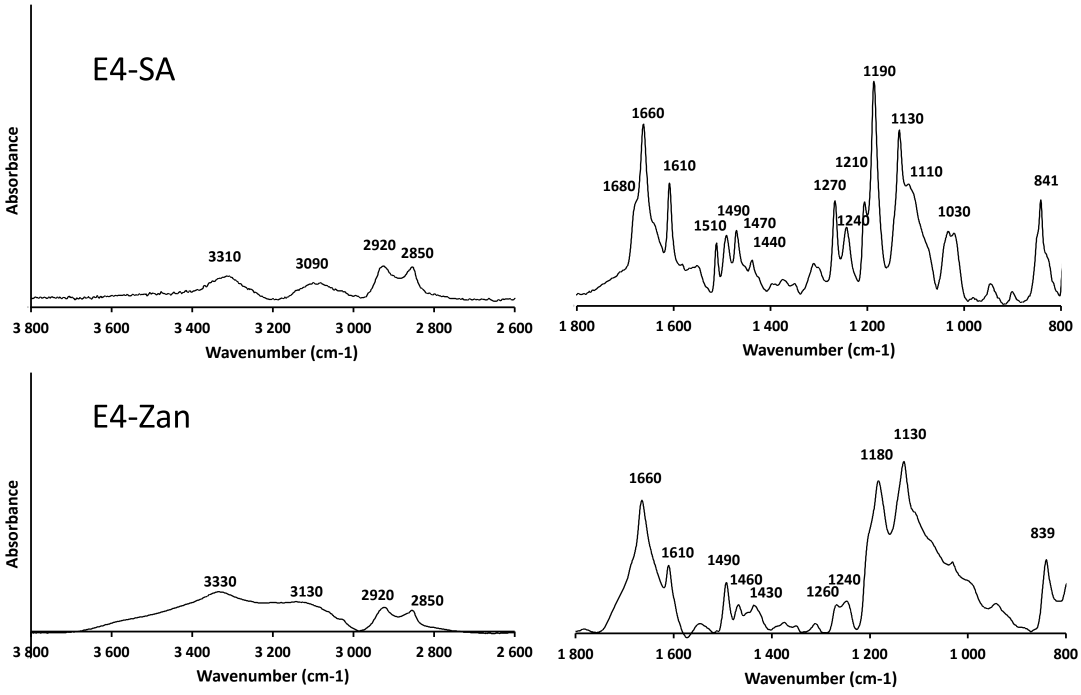

Figure S1. Baseline-corrected high (left) and low (right) frequency regions of IRAS spectra of (A) rSAMs ($\chi_{E4\text{-}SA}$ = 0.15) after assembly in HEPES buffer (10 mM, pH 8) in presence of different molar ratios of E4-Zan and (B) transmission spectra of E4-SA and E4-Zan.

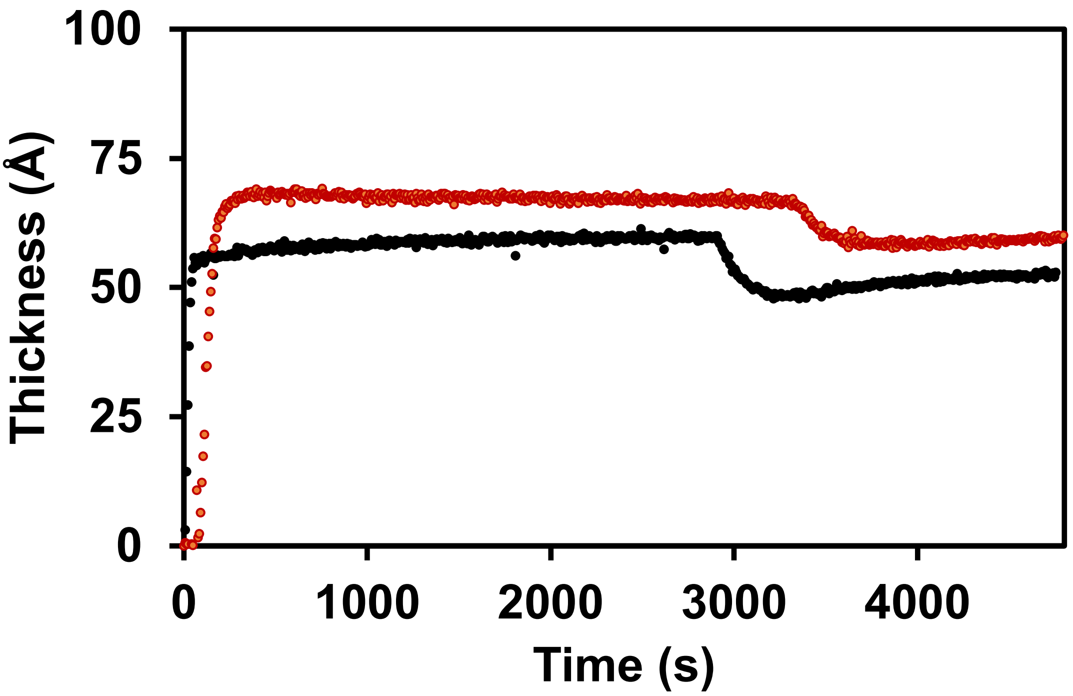

Figure S2. Real time change in film thickness upon immersion of MBA-modified gold substrates in solutions of E4-Zan (red trace) and E4-SA (black trace) in HEPES buffer (10 mM, pH 8). The molar ratios of the ligands were $\chi_{E4\text{-}SA} = \chi_{E4\text{-}SA} = 0.25$ with the rest made up by filler E2-OH. The limiting thicknesses were estimated to 54±1 Å for E4-SA and 63±1 Å for E4-Zan.

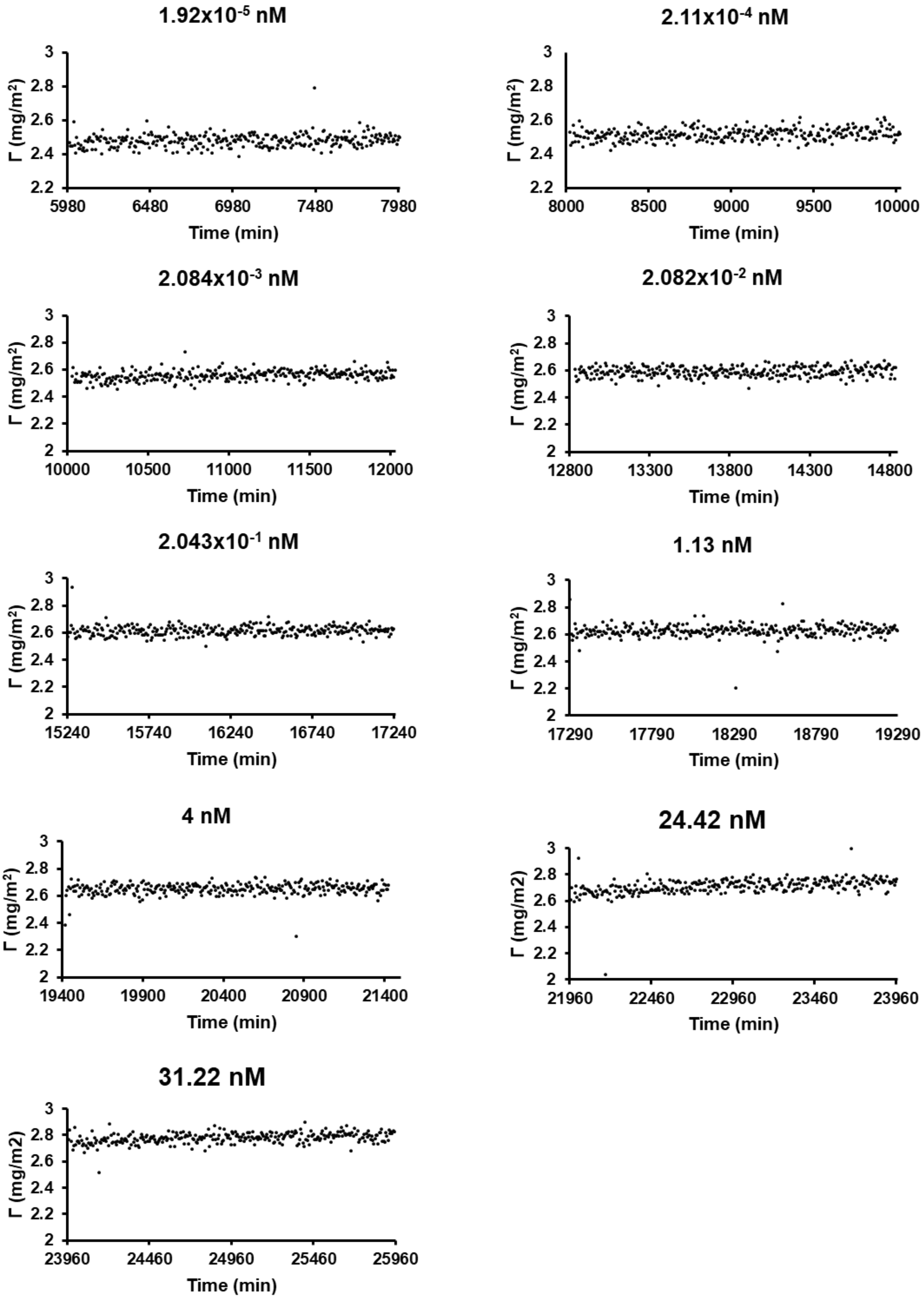

Figure S3. Amount absorbed, (Γ) estimated by *in situ* ellipsometry, versus time on rSAMs χE4-SA = 0.15, χE4-Zan = 0 upon addition incremental amount of N2NA. The final concentration of the N2NA is given on the top.

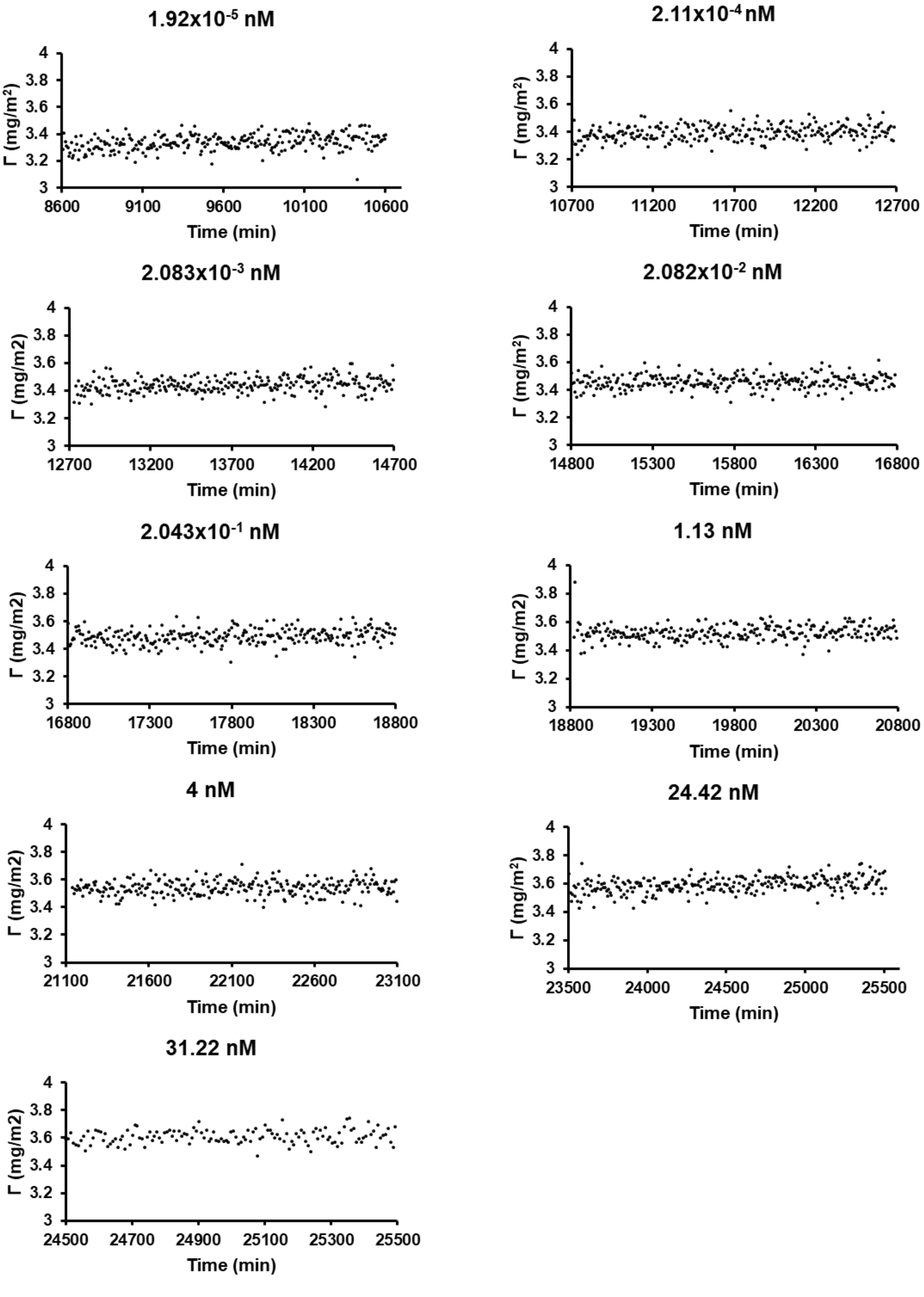

Figure S4. Amount absorbed, (Γ) estimated by *in situ* ellipsometry, versus time on rSAMs χE4-SA = 0.15, χE4-Zan = 0.1 upon addition incremental amount of N2NA. The final concentration of the N2NA is given on the top.

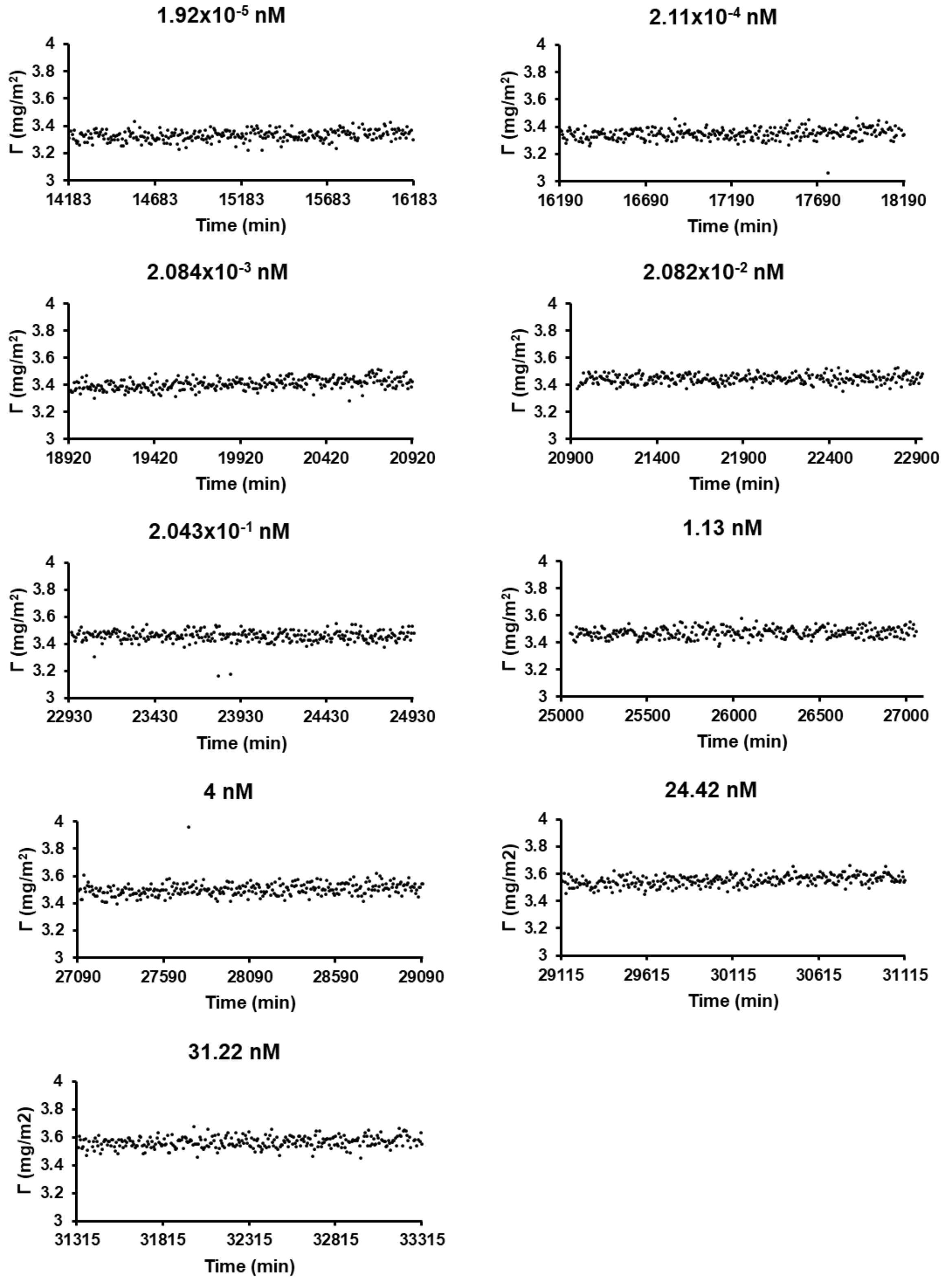

Figure S5. Amount absorbed, (Γ) estimated by *in situ* ellipsometry, versus time on rSAMs χE4-SA = 0.15, χE4-Zan = 0.2 upon addition incremental amount of N2NA. The final concentration of the N2NA is given on the top.

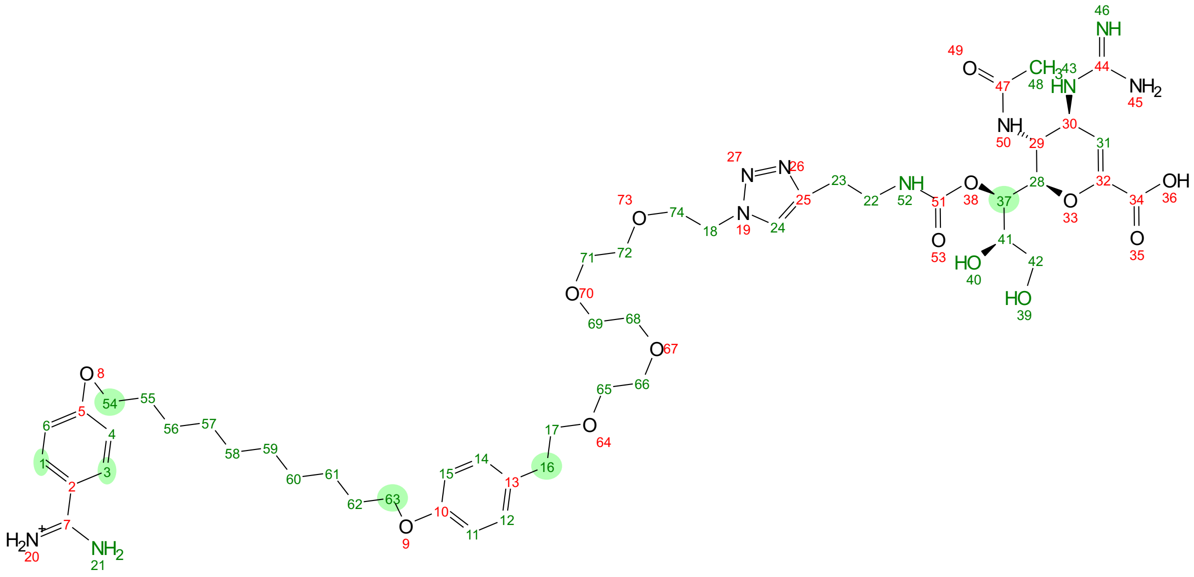

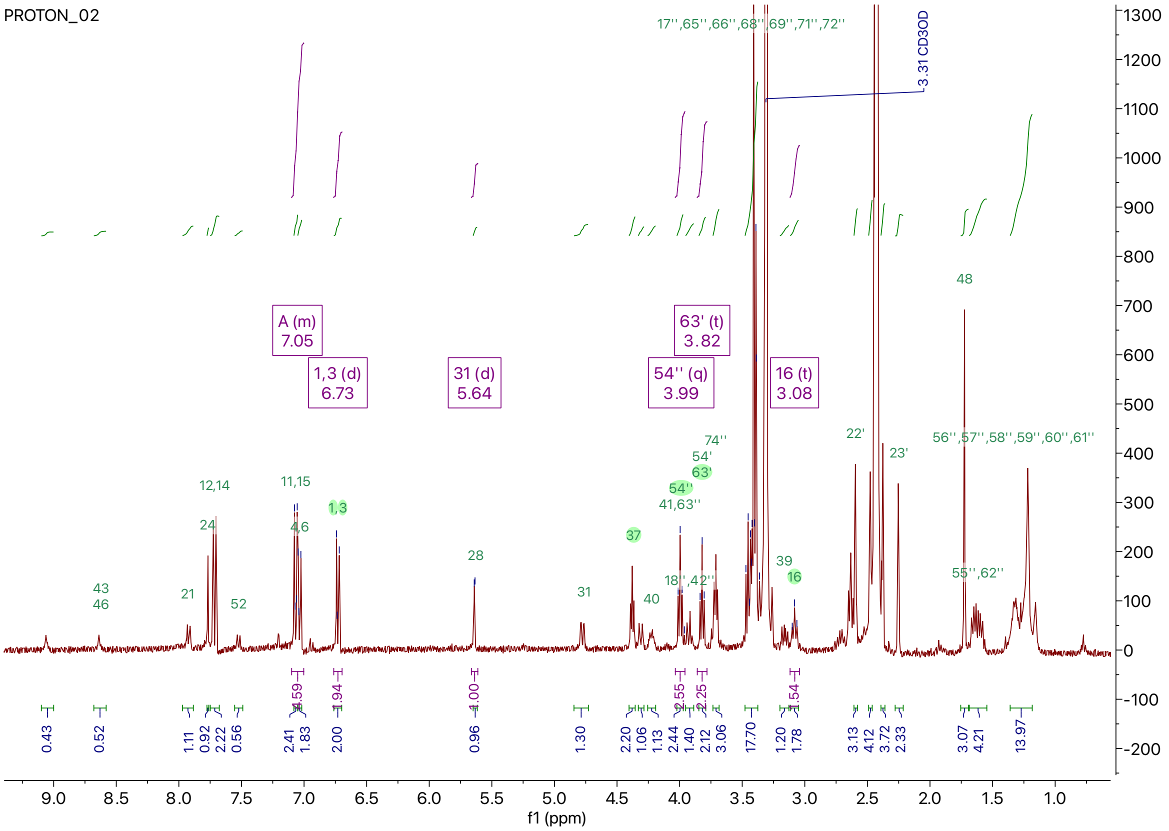

Figure S6. ^{1}H- NMR spectrum of E4-Zan in MeOD after D2O exchange.
